# Broadening the Phenotype Spectrum of *MECP2* Variants in Men

**DOI:** 10.1002/mgg3.70056

**Published:** 2025-01-30

**Authors:** Johannes Lötjönen, Venla Kurra, Hannele Laivuori, Nina Bjelogrlić

**Affiliations:** ^1^ Faculty of Medicine and Health Technology Tampere University Tampere Finland; ^2^ Department of Clinical Genetics Tampere University Hospital Tampere Finland; ^3^ Center for Child, Adolescent and Maternal Health Research, Faculty of Medicine and Health Technology Tampere University Tampere Finland; ^4^ Department of Obstetrics and Gynaecology Tampere University Hospital Tampere Finland; ^5^ Tampere University Hospital Wellbeing Services County of Pirkanmaa Tampere Finland

**Keywords:** exome sequencing, intellectual disability, *MECP2* gene, phenotype, Rett syndrome, X‐chromosomal inheritance

## Abstract

**Background:**

*MECP2* variants cause X‐chromosome‐linked rare developmental syndromes. Typically, the mutation is sporadic, occurs in females and is fatal in men. Accurate genetic and clinical diagnostics are considered essential for the management of symptoms and the development of new treatments. These aims may be difficult to reach before more is known about factors resulting in highly variable clinical pictures among patients carrying the same *MECP2* variant. We describe the clinical picture of two brothers carrying the same *MECP2* variant and compare them with cases published in the literature.

**Methods:**

Most of the *MECP2* mutations are known to be de novo mutations, which is why the recurrence of the mutation in the couple's other children is unlikely. Unexpectedly, our routine genetic testing revealed a 23‐year‐old man (P1) and his younger brother (P2) to carry the same hemizygous pathogenic missense variant c.419C>T, p.(Ala140Val) (transcript NM_004992.3) of *MECP2*, which was found to be inherited from their presumably asymptomatic mother. Thus, further clinical evaluation and comparison with literature cases was considered necessary.

**Results:**

The P1 has a severe syndromic intellectual disorder (ID), whereas his brother has a substantially milder ID predominantly limited to problems in verbal skills. Neither P1 nor his younger brother has been diagnosed with Rett syndrome. The P1 (unlike his younger brother) has several lingual, social and motor difficulties; disruptive behavior was the most difficult symptom to treat. P1's response to several medical and non‐medical treatment trials has remained inadequate, thus requiring the patient to be hospitalised for a long time.

The literature review revealed that apart from our family, there are five other families with more than one male carrying the same *MECP2* p.Ala140Val mutation, such as P1 and P2. The phenotypes of all 24 men from us (*n* = 2) and others (*n* = 22) carrying the same, presumably non‐lethal mutation show great variability.

**Conclusions:**

The p.Ala140Val mutation of *MECP2* in males is associated with a rare X‐chromosomal developmental disorder with highly variable phenotypes. Further studies are needed to better understand all those influencing factors that can explain phenotypic differences within the same genotype to find optimal medicinal therapies.

## Graphical abstract

This case report presents a 23‐year‐old man and his younger brother who both carry maternally inherited hemizygous pathogenic missense variant c.419C>T, p.(Ala140Val) of MECP2. While the index patient has a severe syndromic intellectual disorder (ID), his outpatient brother has substantially milder ID predominantly limited to problems in linguistics skills.

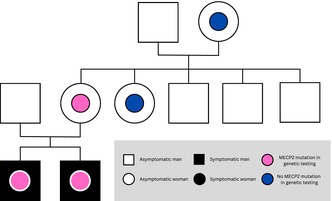

## 1 Background

The clinical symptoms of Rett syndrome and other developmental disorders associated with the *MECP2* gene (OMIM 300055) are known to be highly variable due to multiple pathogenic variants and, on the other hand, the same genotype can have a variable phenotype. The studies on the correlation between genotypes and phenotypes are rare, and they have yielded conflicting results (Wan et al. 1999; Renieri et al. 2003; Chahil, Yelam, and Bollu 2008; Arvio et al. 2021). This makes both specific diagnosis and symptom management challenging, as we illustrate in this paper by our own clinical experience and literature review.

Pathogenic variants in the methyl‐CpG‐binding protein 2 *(MECP2)* gene cause X‐linked developmental disorders. Of these disorders, Rett syndrome is clinically the best known. It is caused in 95%–97% of cases by *MECP2* variants. Originally, Rett syndrome was thought to appear only in females, while in hemizygous males, *MECP2* variants were considered lethal. With the wider use of next‐generation sequencing techniques and exome sequencing becoming a standard tool in the diagnostics of intellectual disorder (ID), the phenotypic diversity of carriers of *MECP2* variants in both genders has broadened. By now, several men with varying phenotypes have been reported (Chahil, Yelam, and Bollu 2008; Amir et al. 1999; Meloni et al. 2000; Shah et al. 2021).

MECP2 mutations leading to highly variable developmental disorders cannot be explained solely by a monogenic dominant X‐chromosomal inheritance pattern (Renieri et al. 2003; Wen et al. 2023). Shah and co‐workers (Shah et al. 2021) have reported the first case of a mosaic MECP2 male who has survived beyond the age of 2 years. Recently, MECP2 mosaicism has been detected in sperm samples from fathers with Rett daughters and healthy men without a family history of Rett syndrome, confirming that germline mosaic mutations can be passed on to offspring (Wen et al. 2023). In addition, the phenotypic heterogeneity associated with MECP2 mutations has been proposed to be due to a digenic inheritance, that is, that a second ‘mutation’ allele in another gene affects the clinical severity of the MECP2 mutation (Renieri et al. 2003).

The clinical phenotypes of the males carrying the MECP2 variants have ranged from severe neonatal encephalopathy to ID with parkinsonism and macro‐orchidism, with partly overlapping features (Table 1) (Chahil, Yelam, and Bollu 2008; Arvio et al. 2021). In this case report, we describe how the same maternally inherited p.Ala140Val mutation (transcript NM_004992.3) in *MECP2* has led to two distinct behavioural symptoms in two intellectually disabled brothers without a clinical diagnosis of Rett syndrome and compare our clinical findings to the cases published in the literature.

**TABLE 1:** Comparison between phenotypes of MECP2 disorders in males described in the literature in comparison to phenotypes observed in our cases P1 and P2.

Phenotype	Feature	Present (%)	P1	P2
*MECP2*‐related severe neonatal encephalopathy	Head growth deceleration/microcephaly	94	−	−
	EEG abnormality	88	−	−
	Hypotonia and/or feeding difficulties infancy	82	+	+
	Severe development delay	82	+	+
	Normal birth parameters	71	+	+
	Movement disorder (e.g., myoclonus, tremors, dystonia)	59	+	−
	Seizures	59	−	−
	Hypertonia of extremities	53	−	−
	Irregular breathing/sleep apnoea	47	−	−
	Poor head control	35	−	−
	GERD	35	+	−
	Mild cerebral atrophy	18	−	−
	Polymicrogyria	6	−	−
Syndromic/nonsyndromic intellectual disability	Severe intellectual disability	90	+	+
	Gait abnormalities	57	+	+
	Poor/absent language skills	47	+	+
	Behavourial problems	40	+	−
	Spasticity	33	+	−
	Hypotonia	23	+	+
	Seizures	20	−	−
	History of regression	17	+	+
	Microcephaly	13	−	−
	Sleep disturbances	13	+	+
	Facial dysmorphism	10	+	+
	Autistic‐like behaviour	3	+	+
Pyramidal signs, parkinsonism & macro‐orchidism (PPM‐X syndrome)	Psychosis	68	NA	−
	Progressive spasticity	68	+	−
	Delayed development	54	+	+
	Intellectual disability	50	+	+
	Speech difficulties	50	+	+
	Pyramidal signs	46	+	−
	Movement disorders	32	+	+
	Macro‐orchidism	19	−	−
	Scoliosis or kyphosis	11	+	+
	Dysmorphic features	5	+	+
	Parkinsonism	3	+	−
	Bilateral juvenile cataract	3	−	−
	Apraxia	3	−	−
	Seizures	3	−	−

Abbreviations: P1, the patient; P2, the patient's brother.

## 2 Case Description

### 2.1 Infancy and Diagnostics

**FIGURE 1:**
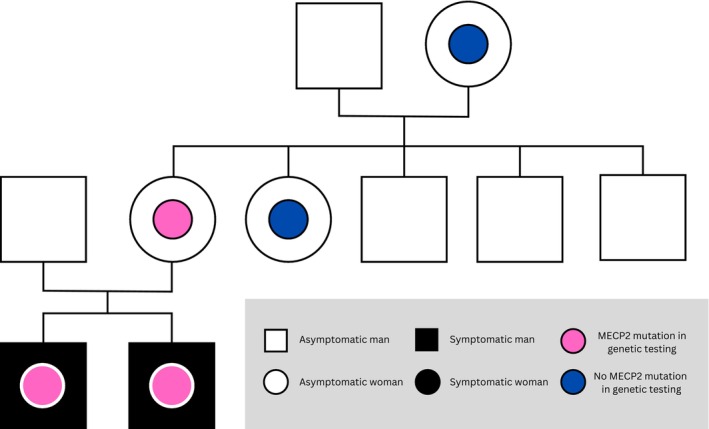
Family tree of P1's and P2's immediate family with P1 and P2 shown as black boxes with red circles in the middle.

The 23‐year‐old index patient (P1) and his three‐year‐old younger brother (P2) are offspring of a healthy non‐consanguineous couple with no previous history of ID in their family. The family tree is illustrated in Figure 1. P1 was born at 39^4/7^ weeks of gestation after an uncomplicated pregnancy with a normal vaginal delivery. The birth measures of P1 were normal: birth weight 3920 g (+1SD), birth length 52 cm (+0.9SD) and head circumference 36 cm (+0.7SD). Parents recall that P1 (unlike his younger brother) had problems with breastfeeding, which they thought to be the first sign of concentration problems. Otherwise, early development was normal.

During the 1st years of life, P1 started to express developmental abnormalities such as restlessness, mood swings and a delay in learning to walk and talk. P1 learned to walk at the age of 1 year and 9 months, and by the end of the 2nd year, his speech consisted of short monosyllabic words.

Conventional investigations of the aetiology of developmental delay were conducted during the 2nd and 3rd years, but the aetiology remained open. Urine metabolic screening, vacuolar cell examination and metabolic screening showed no abnormalities. Also, EEG, brain MRI, vision and auditory testing results were normal. For genetic analysis, DNA was extracted from the peripheral blood sample. Chromosomal microarray and the repeat expansion analysis of the FMR1 gene (fragile X messenger ribonucleoprotein 1) were normal.

At the age of 21, P1's genetic testing was supplemented with exome sequencing during his long‐term rehabilitation in a closed adult unit due to unresponsive, persistent destructive behavior and because his younger brother was known to have a similar but milder ID syndrome. Exome sequencing was performed with Illumina sequencing (Blueprint Genetics laboratory, reference genome GRCh37/hg19), and the variants were classified according to a modified ACMG variant classification system. In exome sequencing, a pathogenic hemizygous c.419>T, p.(Ala140Val) variant of *MECP2* was found in P1. Subsequently, targeted variant testing revealed that the variant was maternally inherited and carried also by P2.

### 2.2 The Current State

#### 2.2.1 Communication

P1 is hypomimic and can only pronounce single monosyllabic or dissyllabic words. Therefore, communication relies on vocalisations, bodily expressions and language aids. However, he can understand speech and follow some simple verbally given orders.

#### 2.2.2 Social Behaviour

P1 is psychologically, socially and emotionally unstable. P1's social state fluctuates from periods of antisocial withdrawal to periods of anxiety, overexcitement and intense attention‐seeking requiring supervision. P1 reacts strongly to other people's behaviour and imitates it. Cognitively, P1 is 3–3.5 years old. Episodically, P1 can be cooperative, calm and well‐behaved when motivated.

#### 2.2.3 Appearance

**P**1 has syndromic features suggestive of genetic aetiology: prominent central face, retrograde small chin, wide mouth, wide gap between teeth and long bushy eyelashes. P1's posture is stooped, pelvis is twisted, and he has mild scoliosis. P1's arches are depressed on both sides, and there is muscle tension in the lower limbs. P1 has a slim build, and his muscle mass is low. P1's peak body mass index was in 2016 (25.2 kg/m^2^), after which he has had unexplained weight loss of up to 5 kg per year, with a minimum body mass index in 2021 (16.7 kg/m^2^). Since then, he has reached a normal BMI of 18.9, with a current weight of 59 kg and a height of 177 cm. Head circumference is normal.

#### 2.2.4 Gross Motor Skills

Since childhood, the movements of P1 have been slow, ataxic and spastic. He was hypotonic as a child, and movement tenderness occurred especially in more challenging motor functions. The patient's gait has several atypical features: low step height, short and slouching step, external rotation of the feet, knees turned into valgus, minimal forward movements and arms crossed tightly in front. The reflexes are symmetrical. His need for help in performing basic daily activities varies.

#### 2.2.5 Fine Motor Skills

After starting the medication, fine motor functions of P1 have been disturbed by a mild fine‐frequency tremor that occurs in the entire upper body, head and lower limbs. Tremors are provoked especially by muscle tension and fatigue. The cogwheel‐like rigidity of the arms hampers fine motor functions, leading easily to falling of things.

#### 2.2.6 Disruptive Behaviour

From the environment's perspective, aggressive behavior toward others is P1's most challenging symptom. The nature of P1's aggression is anxious and unpredictable. Aggression often emerges without a clear trigger. Sometimes, occasional insomnia and breaking of the precise daily structures can provoke aggression.

P1's aggression usually manifests itself as physical and mental violence or as intentional destruction of his own, others' or public property, but still without suicidal features. P1 can cause dangerous situations for himself and others, as he has no sense of danger. P1 has copious bed‐wetting and defecation in the bed. In addition, he has caused himself rectal bleeding by digging in his rectum. P1's laughter is hysterical and inappropriate, and he is hypersensitive to smells. No specific psychiatric diagnosis explaining severe behavioural symptoms could be found in psychiatric consultation.

The variability of P1's behavior has been monitored by the Cohen‐Mansfield agitation inventory (CMAI) questionnaire, the results of which varied from 58 to 144 points during the follow‐up period of 3 years from 2021 to 2023. Yearly averages in the CMAI follow‐up were 75 (2021), 103 (2022) and 104 (2023).

### 2.3 The Current State of the Patient's Brother

The surveillance of P2 has been conducted in an outpatient clinic with no need for specialist consultation or hospitalisation. Therefore, the clinical data of P2 are limited to one appointment (with J.L. and N.B.) and information provided by the parents on his diagnoses and medical history. The birth measurements of P2 had been normal (length 52 cm, weight 4580 g and head circumference 37 cm). Due to hypotonia, he had only learned to walk independently at the age of 28 months. At the age of 21 years, his length is 174 cm, and he weighs 92 kg.

P2's behavioural symptoms have been significantly milder than P1's. The infancy and early childhood of P2 had been similar to that of his brother, both having been hypotonic and having had a significant delay (left at the level of few words) in language development. The father recalls that P2 would have been diagnosed with childhood autism, unspecified intellectual disability and difficulties in speech production. The results of early medical investigations were normal with P2.

P2 is generally calm, well‐behaved and expressively positive. Socially, he is clearly more stable, adaptable and predictable than P1. Despite the learning difficulties, P2 has a normal level of concentration. P2 is obese, he does not have scoliosis, and his posture is normal except for kyphosis. P2 is more skilled than P1 in terms of both fine and gross motor skills. Walking support is needed only for more challenging walking activities. Like P1, P2 has challenges in the coordination of the limb muscles, balance and fine motor skills of the hands. P2 does not have tremors or sensory hypersensitivity. P2 does not exhibit disruptive behavior without a triggering factor.

## 3 Discussion

### 3.1 Diagnostics

The diagnosis of disorders caused by *MECP2* mutations is based on mapping the patient's medical history, a clinical examination and genetic testing (Chahil, Yelam, and Bollu 2008). A patient can be diagnosed with typical Rett syndrome if they meet the following diagnostic criteria: regression, partial or complete loss of learned hand skills and speech, gait disorder and stereotypical hand movements (Neul et al. 2010). A genetic test supports the diagnosis, but it is not necessary in establishing the diagnosis. A pathogenic or likely pathogenic variant in *MECP2* confirms the diagnosis (Mayo Clinic n.d.). Both our case (P1) and his younger brother (P2) fulfilled the genetic criteria for Rett syndrome. The main clinical criteria were met only partly, as they lacked stereotypical hand movements, except that P1 used to clap his hands occasionally when he was excited, when he was angry, or when he wanted something. Supportive criteria indicative of atypical Rett syndrome included hypotonia and scoliosis/kyphosis, which spoke to possible atypical Rett syndrome. However, the clinical features of our two cases cannot be classified under any of the three specific variant forms of Rett syndrome described in 2010 by Neul et al. (2010).

### 3.2 Treatment

There is no curative treatment for the disorders caused by *MECP2* mutations. According to the current recommendations, the primary treatment for Rett's syndrome (as for other *MECP2* related disorders) is a combination of medical and non‐medical multi‐professional treatments. Non‐medicinal forms of treatment include nutritional therapy and personalised rehabilitation therapies.

The multi‐disciplinary care is always adapted to the patient, taking into account the patient's age and the severity of the disorder (Bjeloglic‐Laakso et al. 2014). The treatment is recommended to be started as soon as possible after receiving the diagnosis. The goal of the rehabilitation is to strengthen the patient's motor, sensory, cognitive and social learning and to support the patient's immediate environment psychosocially (Banerjee et al. 2019). Rett's syndrome (like other *MECP2* related disorders) affects several organ systems, but most often symptomatic treatment focuses especially on managing communicational disorders, scoliosis and spasticity (Vashi and Justice 2019).

Due to the instability and severity of symptoms, P1 has had numerous medicinal and non‐medicinal treatment trials since adolescence. His response to multi‐professional and versatile non‐medicinal therapy sessions has remained low. The effectiveness of the medicinal treatment has also been poor, as seen in Figure S1. The difficulties related to medication are displayed in Table S1. The younger brother (P2) has responded well to risperidone well, and medication, at least so far, has not caused any notable side effects. In Table 2 his medication is marked with an asterisk (*).

**TABLE 2:** Characteristics and clinical findings of male patients with maternally inherited p.Ala140Val type described in the literature (selected by the delimitation of Figure S2) in comparison to the patients P1 and P2 described in this case report and the number of symptomatic *MECP2* women in their families.

	Lötjönen	Lambert	Dotti	Klauck	Orrico	Winniepennickx
Age range (years)	20–33	27–67	27–40	11–41	27–40	NA
Degree of ID	1 Severe 1 NA	4 Moderate	4 Severe	6 Moderate	4 Severe	2 Severe 1 Mild 1 NA
Number of symptomatic *MECP2* women in the family (mild symptoms)	0	3	2	4	2	0
Microcephaly	0/2	2/4	0/4	0/6	0/4	NA
Speech difficulties	2/2	4/4	4/4	0/6	4/4	NA
Lively early reflexes	NA	4/4	3/4	6/6	NA	NA
Spasticity/ataxia	2/2	2/4	4/4	6/6	4/4	1/4
Involuntary movements	1/2	1/4	2/4	6/6	4/4	1/4
Behavioural problems	2/2	4/4	NA	4/6	0/4	2/4

### 3.3 MECP2 Genotypes

Next‐generation sequencing has decreased the time needed for accurate molecular diagnosis of patients with rare disorders so significantly that nowadays more and more patients who were previously denied direct sequencing get an explanation for their symptoms (Pascual‐Alonso et al. 2021). Using this method, more than 500 different pathogenic or likely pathogenic variant changes have been found in *MECP2*. The type of mutation has a significant effect on the severity of the disorder, meaning possibly that all variants do not meet conventional clinical diagnostic criteria for Rett syndrome (Banerjee et al. 2019; Vashi and Justice 2019). The p.Ala140Val‐type mutation has never been described in a female with a classical Rett syndrome (Villard 2007). Mutations are most commonly missense, nonsense or frameshift mutations (Chahil, Yelam, and Bollu 2008).

Regardless of the type of mutation, the symptoms of heterozygous women carrying the *MECP2* mutations are often relatively mild. In hemizygous men, the mutation usually leads to either fetal death or a more severe disorder, depending on the type of mutation (Chahil, Yelam, and Bollu 2008). The p.Ala140Val‐type mutation, which was found in our two cases from the same family, is known to cause a milder form of the disorder in men (Arvio et al. 2021).

An ultimate cause of the significant differences observed in the clinical symptoms between P1 and P2 carrying the same maternal *MECP2* variant has remained unclear to us. In general, the severity of the disorder is thought to be influenced by the location of the specific mutation and by the number of mutated cells. According to the literature, *MECP2* can either activate or repress epigenetic transcription (Renieri et al. 2003). As both brothers have inherited the same *MECP2* mutation from their mother, the possibility of mosaicism seems unlikely. Theoretically, the different clinical picture in brothers carrying the same maternal MECP2 p.Ala.140Val mutation might be due to epigenetics or due to digenic inheritance, that is, to another unrecognised gene in P1, which could cause dual molecular diagnosis and explain his severe clinical picture.

### 3.4 Similar Published Cases

In total, 99.5% of *MECP2* mutations are de novo mutations, which is why the recurrence of the mutation in the couple's other children is unlikely (Wan et al. 1999). In addition to the family described in this case report, there are 31 families with hereditary *MECP2* mutations described in the current literature (Zhang et al. 2017; Gutiérrez‐Sánchez et al. 2020). Mutation can be inherited both maternally (*n* = 30) and paternally (*n* = 1). In the Evans, Archer, and Clarke (2006) study, the mutation has been passed to two offspring from a healthy man with mosaicism. Of the families following the maternal inheritance model, three do not have male carriers of the *MECP2* mutation, and in 14 families there is only one male patient with the gene mutation. Of all the families with more than one male offspring with the MECP2 mutation (*n* = 5), the mutation is of the same type p.Ala140Val as in our P1 and P2 cases (see Figure S2) (Zhang et al. 2017). All cases (*n* = 5) reported in the literature that meet the criteria (of the families with > 1 male p.Ala140Val carriers) and our family with two affected males are presented in Table 2.

Lambert et al. (2016) present in their study four men with moderate ID carrying the p.Ala140Val mutation, whose clinical picture resembles that of P1. All patients described in the study have disorders affecting psychomotor and language development, lively early reflexes and mild dysmorphia. Three out of four patients present with lower limb spasticity and inadequate laughing, and half of the patients have microcephaly and short stature. Other symptoms that occur in the family include short stature, difficulty concentrating and abnormal gait. Unlike P1, the character quality of the patients appearing in the study is described as kind, just like that of our P2.

Dotti et al. (2002) present in their study four men with severe ID carrying the p.Ala140Val mutation, all of whom have speech abnormalities and spasticity. Like P1, patients described by Dotti et al. express ataxia and tremors, have no microcephaly or seizures and have no abnormalities detected in MRI or EEG studies.

Orrico et al. (2000) present in their study four men with severe ID carrying the p.Ala140Val mutation, who likewise have speech abnormalities, slowness of movements and rest tremor. In contrast to P1, none of the patients presented in the study had regressed after early development. Klauck et al. (2002) present in their study six similar men with moderate ID carrying the p.Ala140Val mutation, who also present manic‐depressive psychosis. Winniepennickx et al. (2002) present in their study four men carrying the p.Ala140Val mutation, whose ID level had varied from mild to severe. The clinical picture of the patients corresponds to the clinical picture of P1. One of the patients has gynecomastia and hypogonadism, which are not present in P1.

#### 3.4.1 Learning Points

- All *MECP2* variants (known to explain most cases of Rett syndrome) are not lethal in males as thought earlier: the p.Ala140Val mutation is one of those non‐lethal *MECP2* variants. It is good to note that all *MECP2* variants do not necessarily fulfill the clinical diagnostic criteria for Rett syndrome.
- The treatment of diseases caused by *MECP2* variants is challenging due to differences between various gene variants and epigenetic modifications resulting in highly variable clinical symptoms.
- Furthermore, the phenotype can show high variability even within the same pathogenic mutation as demonstrated by the literature review and our cases P1 and P2.
- The clinical consequences of the specific *MECP2* mutations and other influencing factors need to be studied further for the optimisation of individualised treatments.

## Author Contributions

**Johannes Lötjönen:** formal analysis, investigation, data curation, writing original draft preparation, editing the manuscript and visualisation. **Nina** **Bjelogrlić:** conceptualisation, methodology, formal analysis, investigation, resources, commenting and editing the manuscript, supervision and project administration. **Venla Kurra:** investigating, commenting and editing the manuscript. **Hannele Laivuori:** commenting and editing the manuscript.

## Consent

All the individuals concerned have given their written informed consent for the publication of this article. The consent of the patients has been signed by the father.

## Conflicts of Interest

The authors declare no conflicts of interest.

## Institutional Review Board Statement

As a completely retrospective case report, the approval of the institutional review board was not required for this study. The study was conducted according to the guidelines of the Declaration of Helsinki.

## Supporting information


**Figure S1.** Effect of different medications and dose changes to behavioural symptoms as recorded by CMAI questionnaire (Cohen‐Mansfield restlessness scale).

**Figure S2.** Delimitation of the MECP2 cases described in the literature eventually including only the families with motherly inherited p.Ala140Val type mutation and > 1 man with an aberrant MECP2 phenotype.

**Table S1.** The medication of P1 and problems related to pharmacotherapy.

## Data Availability

The data used in this case report are available from the corresponding author upon reasonable request.
