# Supplementary figures and images for: Broadening the Phenotype Spectrum of MECP2 Variants in Men
Source: Mol Genet Genomic Med. 2025 Jan 30;13(2):e70056. doi: 10.1002/mgg3.70056 (PMC11780493; doi:10.1002/mgg3.70056)

# Effect of dosage changes in regular psychiatric medication on CMAI score

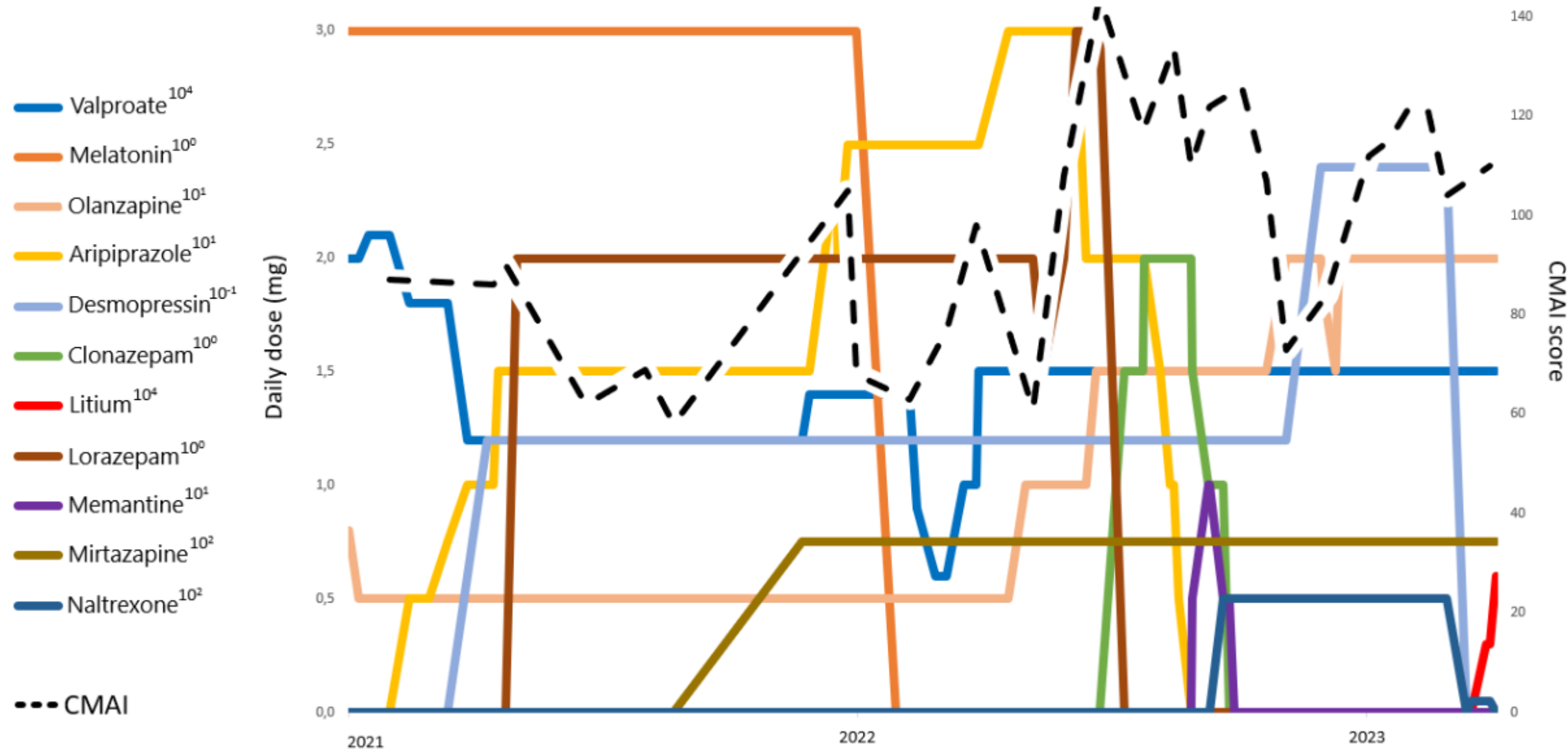

Supplement: Supplementary file 1 — Figure S1. Effect of different medications and dose changes to behavioural symptoms as recorded by CMAI questionnaire (Cohen‐Mansfield restlessness scale). [file MGG3-13-e70056-s002.pdf]

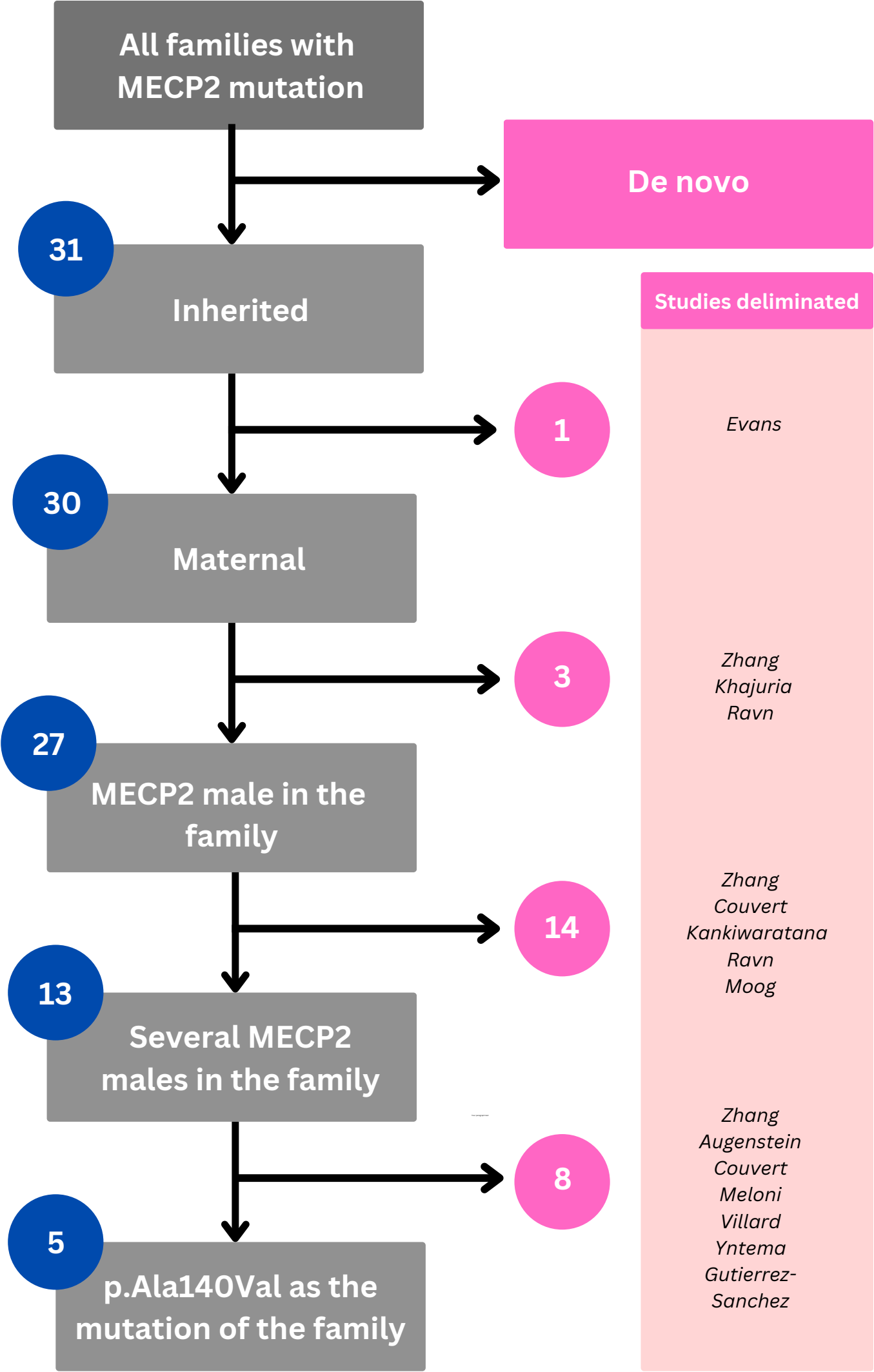

Supplement: Supplementary file 2 — Figure S2. Delimitation of the MECP2 cases described in the literature eventually including only the families with motherly inherited p.Ala140Val type mutation and > 1 man with an aberrant MECP2 phenotype. [file MGG3-13-e70056-s001.pdf]
