# Supplementary material for: Broadening the Phenotype Spectrum of MECP2 Variants in Men
Source: Mol Genet Genomic Med. 2025 Jan 30;13(2):e70056. doi: 10.1002/mgg3.70056 (PMC11780493; doi:10.1002/mgg3.70056)
Supplement: Supplementary file 3 — Table S1. The medication of P1 and problems related to pharmacotherapy. [file MGG3-13-e70056-s003.pdf]

Medication	Degree of use	Problems regarding treatment
**Melatonin***	In use (long term, occasionally paused)	Paradoxical psychic effects
**Risperidone***	In use (long term)	EPS, hyperprolactinaemia, hypotestosteronaemia
**Valproate**	In use (long term)	Hypothyroidism possibly caused by the drug
**Risperidone & Valproate**	Tried	Hypersomnia
**Olanzapine**	In use (long term, occasionally paused)	**Childhood**: fatigue, unresponsiveness to restlessness<br>**Adolescence:** impairment of exercise capacity, urinary incontinence
**Levomepromazine**	In use (long term, occasionally paused)	Dyskinesia, EPS. fatigue
**Clozapine**	In use (recently started)	Unresponsiveness, fatigue
**Methylphenidate**	Tried	Restlessness
**Mirtazapine**	Tried	Unproblematic (left to trial to avoid polypharmacy)
**Aripiprazole**	Tried	Drooling, fatigue, EPS, akathisia
**Quetiapine**	Tried	**Small doses:** rapid increase in drug tolerance<br>**Large doses:** activating effect
**Memantine**	Tried	Mental breakdown, tremor
**Naltrexone**	Tried	Increase in liver values, promoting the development of gallstone disease
**Desmopressin**	Tried	Unresponsiveness
**Lithium**	Tried	Losing the ability to walk
**Calcium folinate**	Tried	Unresponsiveness
**Benzodiatzepines**	Tried when needed)	Bad response or unresponsiveness
**Haloperidol**	When needed	-
**Painkillers**	When needed	-
